# Tailored Predictive Indicators for Weaning Success from High-Flow Nasal Cannula in Postoperative Hypoxemic Patients

**DOI:** 10.3390/life15020312

**Published:** 2025-02-17

**Authors:** Yuh-Chyn Tsai, Shih-Feng Liu, Hui-Chuan Chang, Ching-Min Huang, Wan-Chun Hsieh, Chin-Ling Li, Ting-Lung Lin, Ho-Chang Kuo

**Affiliations:** 1 Department of Respiratory Therapy, Kaohsiung Chang Gung Memorial Hospital, Kaohsiung 833, Taiwan; jane2793@cgmh.org.tw (Y.-C.T.); elaine11142@cgmh.org.tw (H.-C.C.); howmie@cgmh.org.tw (C.-M.H.); jsa102503@cgmh.org.tw (W.-C.H.); musquito16@cgmh.org.tw (C.-L.L.); 2 Division of Pulmonary and Critical Care Medicine, Department of Internal Medicine, Kaohsiung Chang Gung Memorial Hospital, Kaohsiung 833, Taiwan; erickuo48@yahoo.com.tw; 3 Medical Department, College of Medicine, Chang Gung University, Taoyuan 333, Taiwan; 4 Division of General Surgery, Department of Surgery, Kaohsiung Chang Gung Memorial Hospital, Chang Gung University College of Medicine, 123 Ta-Pei Road, Niao-Sung, Kaohsiung 833, Taiwan; tinolin@cgmh.org.tw; 5 Department of Pediatrics, Kaohsiung Chang Gung Memorial Hospital, Kaohsiung 833, Taiwan

**Keywords:** hypoxemic respiratory failure, high-flow nasal cannula, post-surgery

## Abstract

The use of high-flow nasal cannula (HFNC) as an oxygen therapy post-extubation has demonstrated varying success rates across different surgical populations. This study aimed to identify the predictive factors influencing HFNC weaning outcomes in patients with postoperative extubation hypoxemia. We conducted a retrospective analysis of patients in a surgical intensive care unit, categorized into three major postoperative groups: cardiothoracic surgery, upper abdominal surgery, and other surgeries. Our analysis examined pre-extubation weaning profiles, vital signs before and after HFNC initiation, and changes in physiological parameters during HFNC use. A total of 90 patients were included, divided into two groups based on HFNC weaning success or failure. Key parameters analyzed included maximal inspiratory pressure (MIP), PaO_2_/FiO_2_ (P/F) ratio, vital signs, SpO_2_ levels, respiratory rate (RR), heart rate (HR), respiratory rate–oxygenation (ROX) index, and HFNC duration. The findings revealed that cardiothoracic and upper abdominal groups showed significantly higher HFNC weaning success rates (73.3% and 70.6%) compared to the other surgeries group (34.6%) (*p* = 0.004). Critical predictors of successful weaning included pre-HFNC SpO_2_, P/F ratio, and changes in the ROX index, particularly in upper abdominal and other surgeries groups. In cardiothoracic surgery patients, higher maximal inspiratory pressure (MIP) (*p* = 0.031) was associated with improved outcomes, while prolonged HFNC use correlated with weaning success in this group (*p* = 0.047). These findings underscore the necessity of tailoring HFNC strategies to surgical characteristics and individual patient profiles. For cardiothoracic surgery patients, pre-extubation MIP, post-extubation RR, ΔROX, and ΔHR were identified as key predictive factors. In upper abdominal surgery, pre-extubation P/F ratio, post-extubation SpO_2_, and ΔROX played crucial roles. For patients undergoing other types of surgeries, pre-extubation P/F ratio and ΔROX remained the most reliable predictors of HFNC weaning success.

## 1. Introduction

High-flow nasal cannula (HFNC) are a widely used noninvasive respiratory support modality that provides high oxygen flow rates (30–60 L/min) while maintaining a stable temperature and humidity. Unlike conventional oxygen therapy, HFNC offer additional physiological benefits, including improved mucociliary clearance, reduced inspiratory resistance, and enhanced alveolar recruitment. HFNC are more comfortable than noninvasive ventilation (NIV) techniques and have demonstrated greater effectiveness in managing acute hypoxemic respiratory failure post-extubation [1,2,3,4]. HFNC therapy has been shown to effectively reduce respiratory rate (RR), heart rate, dyspnea score, supraclavicular retraction, and thoracoabdominal asynchrony, while also decreasing the need for escalation to invasive mechanical ventilation [5,6]. Additionally, its use is associated with a shorter intensive care unit (ICU) stay and improved oxygenation parameters (SpO_2_ and PaO_2_ levels) [7,8]. Unlike conventional oxygen therapy, HFNC has been recognized as a viable alternative to NIV for preventing post-extubation respiratory deterioration and reducing the need for reintubation [9]. Several systematic reviews and meta-analyses on surgical patients undergoing extubation support the reliable use of HFNC over traditional oxygen therapy and NIV [2,3,10]. However, despite these benefits, HFNC weaning failure remains a significant clinical concern. Unsuccessful HFNC therapy can lead to delayed reintubation, which has been associated with increased morbidity and mortality [11,12]. A recent study highlighted that NIV with active humidification may be superior to HFNC in preventing reintubation, particularly in patients with high-risk factors, such as age above 65, patients with an Acute Physiology and Chronic Health Evaluation II score higher than 12 on extubation day, patients with a body mass index greater than 30, patients with inadequate secretion management, patients experiencing difficult or prolonged weaning, and patients with two or more comorbidities [13].

While HFNC demonstrates clear advantages over standard oxygen therapy and NIV, precision in its initiation, duration, and discontinuation remains crucial [14]. Most existing studies on HFNC have focused on internal medicine patients, leaving limited research on its application in postoperative surgical populations. A 2020 systematic review suggested that the use of HFNC following cardiothoracic surgery holds a moderate level of certainty in improving postoperative respiratory outcomes [15]. Moreover, evidence suggests that heart rate (HR) and the respiratory rate–oxygenation (ROX) index may serve as objective predictors of HFNC weaning success [16]. Notably, ΔHR (change in heart rate) has been identified as a key parameter associated with HFNC outcomes, supporting its role in clinical decision making and individualized respiratory care [17].

Given the increasing application of HFNC in postoperative hypoxemic respiratory failure, it is essential to establish clear prognostic indicators for weaning success and failure. This study aims to identify the key predictors of HFNC weaning success in postoperative patients with extubation hypoxemia, enabling more tailored respiratory management strategies to improve patient outcomes and reduce the risk of delayed reintubation.

## 2. Materials and Methods

### 2.1. Study Design

In this retrospective study, patients were categorized into three groups based on the type of surgery: cardiothoracic surgery, upper abdominal surgery, and other surgeries. The cardiothoracic surgery group primarily consisted of patients from cardiothoracic surgery, with the majority undergoing aortic dissection surgery at Kaohsiung Chang Gung Memorial Hospital in Taiwan. The upper abdominal surgery group included patients undergoing upper abdominal procedures such as liver segment resection and exploratory laparotomy. The other surgeries group consisted of patients who underwent orthopedic, urological, rectal, and plastic surgeries. Data collection focused on postoperative patients who were intubated and admitted to the ICU. After ventilator weaning training, clinical parameters—including arterial blood gas (ABG) tests, laboratory data, Richmond Agitation–Sedation Scale (RASS) scores [18], Rapid Shallow Breathing Index (RSBI), and Maximum Inspiratory Pressure (MIP)—were assessed to determine extubation readiness. Extubation was only performed if patients met clinical criteria and, at the time of extubation, none of the patients were receiving opioid analgesia or other respiratory depressant medications. Those who developed hypoxemia post-extubation hypoxemia were initiated on HFNC therapy and stratified into two groups: 1. Successful HFNC Weaning Group—patients who successfully transitioned from HFNC to conventional oxygen therapy—and 2. Unsuccessful HFNC Weaning Group—patients who required reintubation or prolonged HFNC support beyond 21 days. The outcomes of HFNC weaning were compared between these two groups.

### 2.2. HFNC Therapy Criteria and Protocol

Before considering HFNC, all patients initially received conventional oxygen therapy (e.g., nasal cannula, Venturi mask). HFNC was initiated only if oxygen requirements exceeded the levels achievable with standard high-flow oxygen therapy or if patients exhibited persistent oxygen desaturation (SpO_2_ < 92%), increasing oxygen demand, and clinical signs of respiratory distress.

According to Taiwan National Health Insurance (NHI) reimbursement guidelines, HFNC therapy is restricted to hospitalized adult patients with acute hypoxemic respiratory failure, provided they meet all of the following criteria:

(a) Oxygen therapy ≥ 10 L/min for at least 15 min, with a PaO_2_/FiO_2_ (P/F) ratio ≤ 300.
(b) RR > 25 breaths/min, accompanied by dyspnea or respiratory distress.
(c) Arterial carbon dioxide partial pressure (PaCO_2_) ≤ 45 mmHg.

If the patient met these criteria while on conventional oxygen therapy, HFNC was initiated.

For patients receiving HFNC, vital signs and physiological parameters were systematically recorded at baseline and throughout HFNC therapy. Noninvasive physiological monitoring data were also incorporated to evaluate their predictive value for HFNC weaning success, including weaning profiles prior to extubation, vital signs post-extubation, and dynamic changes in respiratory parameters during HFNC use. These findings confirmed the utility of these clinical markers in predicting HFNC weaning success.

### 2.3. Study Patients and Inclusion Criteria

**Figure 1:**
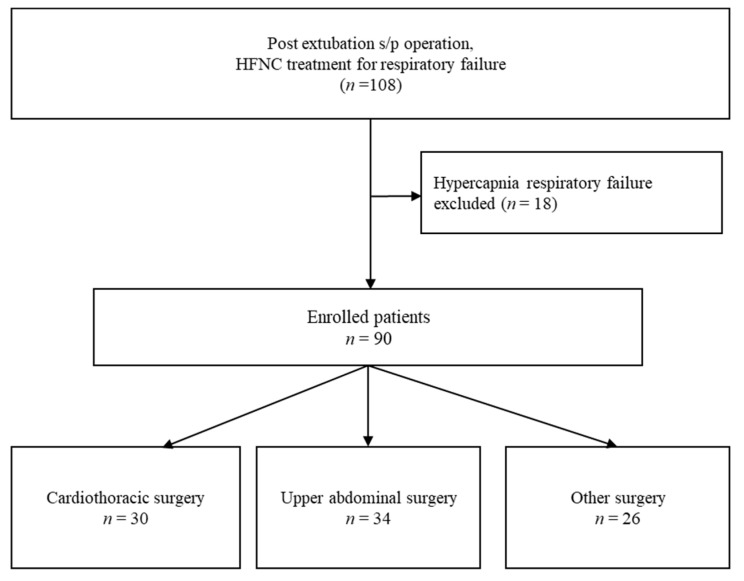
Flowchart of study design and patient selection.

This retrospective analysis included patients with post-extubation hypoxemia requiring HFNC therapy, admitted to the surgical ICU at Kaohsiung Chang Gung Memorial Hospital in Taiwan. Data were collected from 20 October 2016 to 30 December 2018. A total of 108 patients initially met the inclusion criteria, but 18 patients with hypercapnic respiratory failure were excluded, resulting in a final study cohort of 90 patients, categorized into three surgical groups: cardiothoracic surgery, upper abdominal surgery, and other surgeries (Figure 1).

### 2.4. Outcome Measures and Definitions

In this study, the effects of HFNC following surgery were investigated. Both groups were subjected to a retrospective analysis. Patients who required reintubation within 48 h after extubation were classified as experiencing weaning failure. Additionally, those with a prolonged ICU stay exceeding 21 days were also categorized within the weaning failure group, whereas patients who were no longer dependent on HFNC and who subsequently received oxygen therapy (e.g., via nasal cannula or Venturi mask) were regarded as the successful HFNC weaning group. The HR, blood pressure, mean arterial pressure (MAP), SpO_2_, PaO_2_, and PaO_2_/FiO_2_ (P/F) ratios of the two groups were then observed.

### 2.5. Ethical Approval and Patient Informed Consent

This study was approved by the Institutional Review Board (IRB) of Chang Gung Medical Center (IRB No: 201900842B0C501). In accordance with the Declaration of Helsinki and good clinical practice guidelines, the IRB waived the requirement for individual patient consent, as this was a retrospective medical record review.

### 2.6. Statistical Analysis

All statistical analyses were performed using STATA Version 12 (College Station, TX, USA). Continuous variables are expressed as mean ± standard deviation (SD), while categorical variables are presented as absolute values and percentages. Group comparisons were conducted using the Chi-squared test for categorical data and the *t*-test for continuous data. A stepwise multivariate logistic regression analysis was performed to identify predictors of HFNC weaning success and failure, with significance levels set at 0.05 for removal and 0.01 for addition. Model fit was evaluated using pseudo-R² statistics, and the area under the receiver operating characteristic (ROC) curve (AUC) was calculated to assess predictive accuracy, with AUC values interpreted as follows: 0.7–0.8 indicating acceptable discrimination, 0.8–0.9 indicating excellent discrimination, and 0.9–1.0 indicating outstanding discrimination [19].

## 3. Results

### 3.1. Patient Demographics and Surgical Group Distribution

Table 1 presents the respiratory and demographic characteristics among patients categorized into three surgical groups: cardiothoracic surgery (33.33%), upper abdominal surgery (37.78%), and other surgeries (28.89%). The sex distribution showed a predominance of males in the cardiothoracic (70%) and upper abdominal surgery groups (58.8%), while females were more common in the other surgeries group (61.5%). Although this trend approached significance, it did not reach statistical relevance (*p* = 0.057). Patients in the other surgeries group were older on average (77.92 ± 9.85 years) compared to those in the cardiothoracic (65.7 ± 12.06 years) and upper abdominal surgery groups (70.21 ± 14.90 years), although the difference was not statistically significant (*p* = 0.096). The difference in the BMI was not statistically significant (*p* = 0.808). The Charlson Comorbidity Index (CCI) was higher in the upper abdominal (7.35 ± 2.67) and other surgeries groups (7.73 ± 2.03) than in the cardiothoracic surgery group (5.67 ± 2.55), but this difference did not reach statistical significance (*p* = 0.342). The prevalence of COPD across the three groups was 7.1%, 11.82%, and 16.3%, with a *p*-value of 0.578, indicating no statistically significant difference among the groups (*p* = 0.578), the prevalence of chronic heart failure was 20%, 7.12%, and 14.1%, with a *p*-value of 0.237 (*p* = 0.237), suggesting that the differences observed between the groups were not statistically significant.

### 3.2. HFNC Weaning Success Rates

Patients in the cardiothoracic and upper abdominal surgery groups showed significantly higher rates of successful weaning from HFNC at 73.3% and 70.6%, respectively, compared to only 34.6% in the other surgeries group (*p* = 0.004).

### 3.3. Respiratory Mechanics and Oxygenation Parameters

Weaning profiles revealed differences in respiratory mechanics, with the cardiothoracic surgery group achieving a higher maximal inspiratory pressure (MIP) of 37.2 ± 7.1 cm H_2_O compared to 34.24 ± 13.78 cm H_2_O in the upper abdominal group and 31.5 ± 13.49 cm H_2_O in the other surgeries group (*p* = 0.031). However, there were no significant differences in the Rapid Shallow Breathing Index (RSBI) or maximal expiratory pressure (MEP) across groups. The P/F ratio demonstrated a significant difference (*p* = 0.003), with higher values observed in the cardiothoracic and upper abdominal surgery groups, suggesting better oxygenation profiles. However, no significant differences were observed in the Rapid Shallow Breathing Index (RSBI) or maximal expiratory pressure (MEP) across the groups. These findings underscore the impact of surgical type on respiratory mechanics and HFNC weaning outcomes, highlighting the need for tailored respiratory support strategies based on surgical characteristics.

### 3.4. Comparison of HFNC Weaning Success and Failure

Table 2 presents a detailed comparison between successful and unsuccessful HFNC weaning groups—in the cardiothoracic surgery group (*p* = 0.003), with higher values in the success group (39.64 ± 5.63) compared to the failure group (30.5 ± 8.99). In the upper abdominal surgery group, no significant difference was found (*p* = 0.662), as both success and failure groups showed nearly identical values (34.92 ± 13.78 vs. 34.92 ± 14.39). Similarly, for other surgeries, no significant difference was noted (*p* = 0.502), although the success group had slightly higher values (34 ± 13.42 vs. 30.18 ± 13.74). Pre-HFNC FiO_2_ levels revealed notable differences across surgery types. For cardiothoracic surgery, the mean FiO_2_ was 38.5 ± 13.63 in the success group and 46.88 ± 11.32 in the failure group, with no significant difference (*p* = 0.658). In upper abdominal surgery, the success group had a mean FiO2 of 32.5 ± 2.77, while the failure group had a significantly higher mean of 73 ± 29.77 (*p* = 0.00 **). Similarly, for other surgeries, the success group had a mean FiO_2_ of 33.78 ± 3.31, compared to 50.42 ± 25.43 in the failure group, showing a significant difference (*p* = 0.00 **). SpO_2_ levels were significantly different in upper abdominal surgery patients, suggesting SpO_2_ may be a potential predictor of HFNC weaning success (*p* = 0.007).

### 3.5. ROX Index and Other Predictors of HFNC Weaning

Respiratory rate (RR) is an important factor influencing the success of HFNC weaning. The RR was significantly lower in the successful weaning group compared to the unsuccessful group (*p* = 0.045). Additionally, the change in ROX index before and after HFNC use increased by 2.72 (±8.33) in the successful group versus 0.06 (±3.77) in the unsuccessful group (*p* = 0.031). The duration of HFNC use was longer in the successful group than in the unsuccessful group (*p* = 0.002).

Following extubation after upper abdominal surgery, both pre-HFNC, SpO_2_, and the PaO_2_/FiO_2_ (P/F) ratio were significant predictors of successful HFNC weaning. In the successful group, SpO2 and P/F ratios were higher than those in the unsuccessful group, with values of 96.42 (±1.55) vs. 92.73 (±1.55) (*p* = 0.007), and 289.23 (±97.97) vs. 170.28 (±38.95), with *p* = 0.006, respectively. In terms of respiratory rate, there was a decrease in the successful group and an increase in the unsuccessful group before and after HFNC use (*p* = 0.036).

In other surgical patients, significant differences were observed in pre-HFNC SpO_2_, SpO_2_ before and after HFNC treatment, ROX index, and the duration of HFNC use. SpO_2_ increased in the successful group compared to the unsuccessful group (*p* = 0.001), and the ROX index also showed an increase before and after HFNC use in the successful group (*p* = 0.020). Finally, the number of HFNC days was fewer in the successful weaning group compared to the unsuccessful group (*p* = 0.001).

### 3.6. Predictive Modeling of HFNC Weaning Success

**Figure 2:**
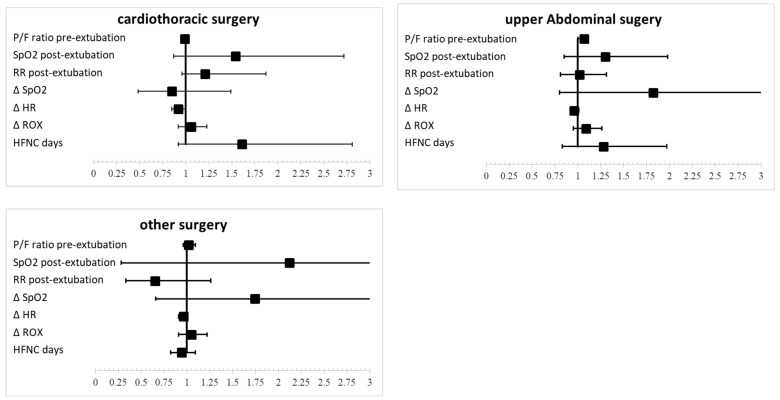
Adjusted odds ratios and 95% confidence intervals for the likelihood of successful weaning from high-flow nasal cannula (HFNC) across different surgical procedures, taking into account vital sign factors.

Table 3 and Figure 2 The table summarizes the adjusted odds ratios (ORs) and 95% confidence intervals (CIs) for successful weaning from high-flow nasal cannula (HFNC) across cardiothoracic, upper abdominal, and other surgeries groups. The pre-extubation P/F ratio was significant only in the upper abdominal surgery group (OR 1.07, *p* = 0.044). For SpO_2_ post-extubation HFNC, ORs were 1.54 (*p* = 0.136) for cardiothoracic, 1.30 (*p* = 0.222) for upper abdominal, and 2.12 (*p* = 0.468) for other surgeries groups, indicating no significant associations. RR had an OR of 1.21 (*p* = 0.042) in the cardiothoracic surgery group, suggesting decreased likelihood of weaning with higher RR, while results were not significant for other surgeries. The pre-extubation P/F ratio was significant only in the upper abdominal surgery group (OR 1.07, *p* = 0.044). The change in SpO_2_ showed no significant results, whereas delta heart rate had an OR of 0.92 (*p* = 0.007) in the cardiothoracic surgery group, indicating reduced likelihood of weaning with increased heart rate. Delta ROX showed positive associations, with ORs of 1.06 (*p* = 0.025) in the cardiothoracic and 1.05 (*p* = 0.014) in other surgeries groups. HFNC duration (the duration of HFNC therapy prior to weaning) was significant in the cardiothoracic surgery group (OR 1.61, *p* = 0.047), suggesting that longer HFNC may correlate with successful weaning.

### 3.7. ROC Curve Analysis

**Figure 3:**
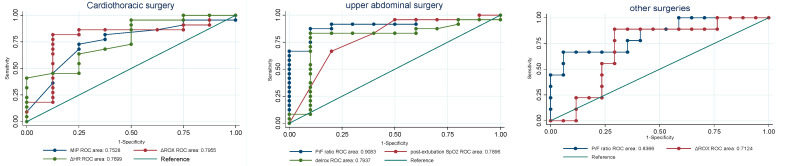
ROC curves for predicting HFNC weaning success in cardiothoracic surgery, upper abdominal surgery, and other surgeries.

Figure 3 presents the ROC curve analysis, revealing variability in predictive parameters across different surgical groups; cardiothoracic surgery, ΔROX (AUC = 0.7955), and ΔHR (AUC = 0.7659) demonstrated the highest predictive accuracy, while pre-extubation MIP (AUC = 0.7528) also contributed significantly. In upper abdominal surgery, the pre-extubation P/F ratio (AUC = 0.9063) emerged as the most reliable predictor, followed by ΔROX (AUC = 0.7937) and post-extubation SpO_2_ (AUC = 0.7896), highlighting the importance of oxygenation parameters. For other surgeries, the pre-extubation P/F ratio (AUC = 0.8366) was the strongest predictor, with ΔROX (AUC = 0.7124) showing moderate predictive value. These findings underscore the need for tailored prediction models that account for the unique characteristics of each surgical population.

Key insights from this study highlight that predictive parameters for HFNC weaning success vary across surgical groups. For cardiothoracic surgery, pre-extubation MIP, post-extubation RR, ΔROX, and ΔHR were identified as critical predictors. In upper abdominal surgery, key predictors included the pre-extubation P/F ratio, post-extubation SpO_2_ and ΔROX. For patients undergoing other surgeries, the pre-extubation P/F ratio and ΔROX emerged as the most consistent and reliable predictors, underscoring their relevance across different surgical categories. These findings emphasize the importance of developing tailored prediction models that incorporate these specific parameters for each surgical group. Refer to Figure 3 for a comprehensive visualization of these results.

## 4. Discussion

This study highlights the significant impact of surgical type on HFNC weaning success. Patients undergoing cardiothoracic and upper abdominal surgeries exhibited significantly higher weaning success rates (73.3% and 70.6%) compared to those in the other surgeries group (34.6%). This variation suggests that surgical characteristics and respiratory mechanics play a critical role in HFNC outcomes.

### 4.1. Predictors of HFNC Weaning Success and Failure

Our findings align with previous research indicating that pre- and postoperative inspiratory muscle training is beneficial for patients undergoing cardiothoracic surgery [20]. Higher maximal inspiratory pressure (MIP) and better respiratory mechanics may explain the higher HFNC success rates in this group. Conversely, patients in the “other surgeries” group demonstrated poorer HFNC outcomes, likely due to underlying pulmonary impairment, older age, and higher comorbidity burden.

### 4.2. The Role of ROX Index in HFNC Weaning

This study confirmed that tracking ROX index trends before and after HFNC use serves as a valuable predictive tool. A declining ROX index, coupled with clinical signs of worsening vital signs, should prompt early intervention, including consideration for intubation, rather than prolonged HFNC therapy. These findings emphasize the importance of monitoring dynamic changes in the ROX index to guide HFNC management strategies.

Our results align with previous studies that suggest routine HFNC use after cardiac surgery may not significantly improve the SpO_2_/FiO_2_ ratio by postoperative day 3, but instead reduces the need for increased respiratory support [21]. In this study, the ROX index remained a meaningful predictor before and after HFNC use, reinforcing its clinical utility. Although the SpO_2_/FiO_2_ ratio did not significantly increase, RR decreased, leading to an improved ROX index, which correlates with prior research findings [22].

### 4.3. HFNC Efficacy in Upper Abdominal Surgery

In upper abdominal surgery, evidence suggests that HFNC is more efficient than a simple oxygen face mask in reducing lung atelectasis, improving oxygenation, decreasing the respiratory rate, and shortening ICU and hospital stays [23]. Our findings indicate that patients with a P/F ratio < 170 and SpO_2_ < 93% had poor HFNC outcomes, suggesting that HFNC should not be prolonged in these cases. Instead, early reintubation should be considered to prevent deterioration.

Prior research has shown that if HFNC is used for up to 12 h in patients with hypoxemic respiratory failure and the ROX index remains above 4.88, reintubation can often be avoided [24]. Our study reinforces this threshold, supporting its potential use in clinical decision making.

### 4.4. Clinical Implications of ROX Index and HFNC Weaning

One of the most significant findings in this study was that the change in ROX index before and after HFNC was strongly correlated with HFNC weaning success. The successful weaning group showed an increasing ROX index, while the failure group showed a decline—a statistically significant difference. This aligns with previous systematic reviews suggesting that ROX index trends may serve as a moderate predictor of HFNC failure risk [25]. Our study also explored other key physiological markers, such as HR and RR, which were lower in patients who successfully weaned from HFNC. The ROX-HR index has been proposed as a promising tool for early identification of HFNC failure, and our findings support its potential clinical application in predicting treatment success [16].

In addition, the P/F ratio’s large standard deviations suggest substantial variability in oxygenation impairment among patients within each surgical group, likely attributable to differences in baseline lung function, the physiological impact of surgery, or pre-existing conditions. Although the *p*-value indicates a statistically significant difference between groups, the clinical relevance of this finding should be interpreted with caution due to the high degree of variability. The statistics in the descriptive analysis showed meaningful differences between successful and unsuccessful HFNC weaning groups across all surgical categories: cardiothoracic surgery (266 vs. 233), upper abdominal surgery (289 vs. 170), and other surgeries (292 vs. 221). These findings align with previous studies recommending the use of HFNC at a P/F ratio of 200–300 as an effective range for oxygenation support. However, when the P/F ratio falls below 200, the use of noninvasive ventilation (NIV) is strongly advised to prevent delayed intubation and ensure timely intervention and treatment [26]. In addition to the aforementioned parameters, both the SpO_2_ level and the HR, which are easily monitored, can also serve as reliable predictors of HFNC weaning success. Using these simple vital signs enables timely treatment adjustments and helps avoid delays. These conditional recommendations provide valuable guidance for clinicians in selecting the most appropriate form of noninvasive respiratory support for patients in various acute care settings [27]. This retrospective study offers important insights and serves as a practical reference for clinical applications.

This study has several limitations. First, as a retrospective study, the relatively small sample size may limit generalizability, highlighting the need for larger cohorts in future research to ensure more robust statistical validation. Second, post-extubation arterial blood gas (ABG) monitoring was inconsistent; while ABG levels were recorded before extubation, not all patients had post-extubation ABGs, introducing variability in the dataset. Incorporating additional respiratory pattern indicators, such as work of breathing assessments, could enhance predictive accuracy. Third, the timing of ROX index collection was not standardized, potentially affecting interpretation. Future studies should investigate optimal time intervals for ROX index assessment to improve its predictive utility.

## 5. Conclusions

This study confirms that hypoxemia alone is insufficient to determine HFNC success; other respiratory parameters, such as MIP, RR, and ROX index trends, must also be considered. For cardiothoracic surgery patients, key predictive factors included pre-extubation MIP, post-extubation RR, ΔROX, and ΔHR, whereas for upper abdominal surgery patients, the pre-extubation P/F ratio and ΔROX were the strongest predictors. In patients undergoing other types of surgeries, ΔROX remained the most consistent predictor, highlighting its broad clinical applicability. Furthermore, this study underscores the value of noninvasive physiological monitoring in predicting HFNC weaning success, suggesting that predictive evaluations should be tailored based on surgical type; cardiothoracic patients may benefit from early assessments, while upper abdominal and other surgical groups may require more nuanced monitoring. Developing tailored prediction models that incorporate specific surgical characteristics is essential for enhancing HFNC weaning accuracy and improving patient outcomes.

## Figures and Tables

**Table 1:** The baseline characteristics of patients with hypoxemic respiratory failure across different surgical types.

	Cardiothoracic Surgery	Upper Abdominal Surgery	Other Surgeries	*p* Value
observation	30 (33.33%)	34 (37.78%)	26 (28.89)	
Sex				0.057
Male	21 (70%)	20 (58.8%)	10 (38.5%)	
female	9 (30%)	14 (41.2%)	16 (61.5%)	
Age	65.7 (12.06)	70.21 (14.90)	77.92 (9.85)	0.096
CCI	5.67 (2.55)	7.35 (2.67)	7.73 (2.03)	0.342
successful weaning from HFNC	73.3%	70.6%	34.6%	0.004
Weaning profile before extubation				
RSBI	71.3 (38.11)	76.15 (51.91)	80.89 (50.8)	0.204
MIP	37.2 (7.71)	34.24 (13.78)	31.5 (13.49)	0.031 *
MEP	28.33 (23.24)	45.68 (26.85)	44.69 (30.42)	0.383
P/F ratio	257.41 (124.77)	254.24 (100.65)	245.97 (61.89)	0.003 **

* *p* < 0.05, ** *p* < 0.01.

**Table 2:** Comparison of the noninvasive monitoring parameters of the patients with hypoxemic respiratory failure after surgery in the successful and failed high-flow nasal cannula weaning groups.

		Cardiothoracic Surgery Mean (± SD)			Upper Abdominal Surgery Mean (± SD)			Other Surgeries Mean (± SD)		
		Success	Failure	*p* Value	Success	Failure	*p* Value	Success	Failure	*p* Value
Weaning profile	MIP	39.64 (5.63)	30.5 (8.99)	0.003 **	34.92 (13.78)	34.92 (14.39)	0.662	34 (13.42)	30.18 (13.74)	0.502
Pre-HFNC	FiO_2_	38.5 (13.63)	46.88 (11.32)	0.658	32.5 (2.77)	73 (29.77)	0.00 **	33.78 (3.31)	50.42 (25.43)	0.00 **
	SpO_2_	96.60 (2.61)	94.9 (3.37)	0.129	96.42 (1.55)	92.73 (1.55)	0.007 **	97.11 (1.40)	95.48 (2.11)	0.219
	RR	21.81 (6.45)	24.62 (3.11)	0.045 *	21.63 (6.72)	25.5 (5.85)	0.663	23 (4.48)	23.64 (5.35)	0.576
	HR	97.32 (15.70)	91.13 (17.59)	0.716	100.5 (17.89)	109.9 (18.22)	0.947	96.56 (13.59)	106.53 (18.47)	0.347
	MAP	86.25 (15.85)	84.25 (15.63)	0.457	92.40 (14.94)	86.29 (14.35)	0.887	86.07 (13.24)	86.07 (11.34)	0.628
	P/f ratio	266.24 (112.57)	233.15 (159.89)	0.243	289.23 (97.97)	170.28 (38.95)	0.006 **	292.25 (49.23)	221.47 (54.14)	0.765
	ROX	13.39 (4.58)	10.31 (3.38)	0.367	14.41 (5.32)	10.34 (4.00)	0.336	12.82 (2.05)	11.44 (3.19)	0.181
Delta (Δ)	SpO_2_	1.32 (2.0)	2 (2.87)	0.247	1.75 (1.98)	0.9 (3.21)	0.073	1.45 (2.01)	−1.11 (7.56)	0.001 **
	RR	−3.5 (7.14)	−1.62 (4.90)	0.256	−2.7 (7.42)	1.46 (7.21)	0.036 *	−2 (6.56)	−0.24 (8.15)	0.499
	HR	−6.41 (12.56)	10.63 (18.35)	0.007 **	−6.3 (15.65)	7.5 (26.30)	0.053	−13.11 (15.44)	−0.77 (18.91)	0.529
	MAP	0.50 (15.09)	5.38 (15.03)	0.991	−4.64 (17.26)	1.54 (19.26)	0.695	−3.52 (23.08)	−5.74 (23.94)	0.907
	ROX	2.72 (8.33)	0.06 (3.77)	0.031 *	−0.3 (5.64)	−2.86 (4.50)	0.442	1.46 (2.97)	−0.12 (6.77)	0.020 **
	HFNC days	5.14 (6.32)	2.38 (1.77)	0.002 **	3.88 (2.07)	3 (2.05)	0.978	5.22 (2.44)	7.59 (8.71)	0.001 **

*: *p* < 0.05, **: *p* < 0.01.

**Table 3:** Stepwise multivariate logistic regression analysis examining the success and failure of HFNC weaning across three types of surgeries: cardiothoracic surgery, upper abdominal surgery, and other surgeries.

	Cardiothoracic Surgery		Upper Abdominal Surgery		Other Surgeries	
	OR (95%CI)	*p* Value	OR (95%CI)	*p* Value	OR (95%CI)	*p* Value
P/F ratio pre-extubation	0.99 (0.98–1.01)	0.423	1.07 (1.00–1.11)	0.044 *	1.02 (0.96–1.09)	0.439
SpO_2_ post-extubation	1.54 (0.87–2.72)	0.136	1.30 (0.85–1.98)	0.222	2.12 (0.28–16.37)	0.468
RR post-extubation	1.21 (0.96–1.87)	0.042 *	1.02 (0.81–1.31)	0.844	0.65 (0.33–1.26)	0.006
ΔSpO_2_	0.85 (0.48–1.49)	0.569	1.82 (0.80–4.13)	0.153	1.74 (0.66–4.57)	0.260
ΔHR	0.92 (0.85–1)	0.007 **	0.96 (0.92–1.01)	0.061	0.96 (0.91–1.01)	0.089
ΔROX	1.06 (0.92–1.23)	0.025 **	1.09 (0.95–1.26)	0.205	1.05 (0.91–1.22)	0.014 **
HFNC days	1.61 (0.92–2.81)	0.047 *	1.28 (0.83–1.97)	0.233	0.94 (0.82–1.09)	0.434

*: *p* < 0.05, **: *p* < 0.01.

## Data Availability

The datasets used during the current study are available from the corresponding author on reasonable request. Data supporting reported results can be obtained on request.

## Abbreviations

HFNC	High-flow nasal cannula
RR	Respiratory rate (number of breaths per minute)
HR	Heart rate (number of heart beats per minute)
P/F ratio	PaO_2_/FiO_2_ ratio (arterial oxygen pressure to the fraction of inspired oxygen)
ROX	Respiratory rate–oxygenation
